# Alcohol‐induced altered glycans in human tracheal epithelial cells promote bacterial adhesion

**DOI:** 10.1002/2211-5463.70188

**Published:** 2025-12-21

**Authors:** Pi‐Wan Cheng, Souvik Datta, Derrick R. Samuelson

**Affiliations:** ^1^ Department of Biochemistry and Molecular Biology, College of Medicine University of Nebraska Medical Center Omaha NE USA; ^2^ Fred & Pamela Buffett Cancer Center University of Nebraska Medical Center Omaha NE USA; ^3^ Department of Internal Medicine, College of Medicine University of Nebraska Medical Center Omaha NE USA

**Keywords:** alcohol toxicity, altered glycans, human tracheal epithelial cells, *Klebsiella pneumoniae*, *Streptococcus pneumoniae*

## Abstract

Heavy alcohol drinking is known to increase the risk of bacterial pneumonia. However, the link between alcohol levels and risk of infection remains underexplored. Recently, we found that alcohol induced α2‐6sialo mucin O‐glycans in human tracheobronchial epithelial cells, which mediated the killing of U937 macrophages. By extending this study, we focus here on whether altered glycans induced by alcohol in human airway epithelial cells can promote adhesion of *Klebsiella pneumoniae* (*Kp*) and *Streptococcus pneumoniae* (*Sp*). We have found that exposure of human tracheal epithelial cells to alcohol also induces high mannose N‐glycans terminated with α3mannose and increases adhesion of *Kp*, which is inhibited by αmethylmannoside or aldehyde dehydrogenase 2 activator 1. Further, the α2‐6sialo mucin O‐glycans induced by alcohol in human tracheal epithelial cells also enhance the adhesion of *Sp*, which is inhibited by ovine submaxillary mucin or aldehyde dehydrogenase 2 activator 1. We conclude that alcohol induces altered glycans in human airway epithelial cells, which increase the risk of bacterial pneumonia by compromising immune function and promoting the adhesion of *Kp* and *Sp*.

## Graphical abstract

Alcohol induces altered glycans to promote bacteria adhesion.

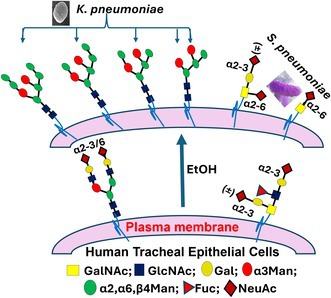

**Abbreviations**
Alda 1: aldehyde dehydrogenase 2 activator 1
ANOVA: analysis of variance
CFU: colony forming units
DisT: disialyl‐T
DUI: driving under the influence
GM130‐GRASP65: GRASP65‐Golgi matrix protein 130‐Golgi reassembly stacking protein 65
GNA: Galanthus nivalis agglutinin
GT: glycosyltransferase
HTE cells: immortalized human tracheal epithelial cells
OSM: ovine submaxillary mucin
Siglec 7: sialic acid‐binding immunoglobulin‐like lectin 7
sLe^a^
: sialyl Lewis a
SNA‐I: Sambucus nigra agglutinin‐I
sT: sialyl‐T
sTn: sialyl‐Tn
T antigen: Galβ3GalNAcαSer/Thr
TACA: tumor associated carbohydrate antigens
Tn: GalNAcαSer/Thr
α‐MM: αmethyl‐D‐mannoside

Alcohol drinking is a common phenomenon in human society. Approximately 2 billion people worldwide drink alcohol regularly, and more than 76 million adults suffer from alcohol use disorder and are unable to control alcohol use [1]. Chronic consumption of excessive alcohol can compromise immune functions and increase the risk of pneumonia [2]. However, the mechanism driving this increased risk remains ill‐defined. Heavy drinkers have an 83% increase in community‐acquired pneumonia compared with nondrinkers and light drinkers [3]. In fact, for every increase of 10–20 g in alcohol consumption, which is equivalent to one serving of a 12 oz beer, per day, there is an increase of 8% in the relative risk of contracting community‐acquired pneumonia [3]. *Streptococcus pneumoniae* (*Sp*) is the leading etiological agent of pneumonia in heavy alcohol users [4]. Yet, many other pathogens, including *Klebsiella pneumoniae* (*Kp*), are also isolated from alcohol‐consuming individuals [5], which has been confirmed in an alcohol‐fed animal model [6].

Glycans are known to play an important role in bacterial infections [7, 8]. For example, sialylated glycans can promote the development of pneumonia by serving as the ligands of the adhesins of pneumonia‐causing bacteria, such as *Sp* [9], and compromise the cytotoxic function of immune cells by serving as the ligands of the inhibitory receptors, such as sialic acid‐binding immunoglobulin‐like lectin 7 (Siglec 7) [10, 11]. An increase in sialylated mucin O‐glycans has been found in the intestinal mucins of alcohol‐fed rats [12, 13]. Recently, we have found that alcohol can cause Golgi fragmentation [14], which led to the formation of not only high mannose N‐glycans in hepatocytes [15] but also α2,6sialyl mucin O‐glycans in human airway epithelial cells [16]. Because α‐mannoside and sialylated mucin O‐glycans can serve as the ligands of the adhesins of *Kp* [17] and *Sp* [9], respectively, we decided to examine whether these altered glycans induced by alcohol in human airway epithelial cells can promote adhesion of *Kp* and *Sp*.

## Materials and methods

The materials used in this study and their suppliers are listed below. All suppliers are located in the United States. *Streptococcus pneumoniae* (*Sp*) (ATCC® BAA‐334™, strain TIGR4 [JNR.7/87], Capsular serotype 4) and *Krebsiella pneumoniae* (*Kp*) (strain 43 816, serotype 2), the American Type Culture Collection, Manassas, VA, USA; Bronchial epithelial cell culture medium (Cat. No. LL‐0023), Lifeline Cell Technology, Frederick, MD, USA; α‐methyl‐D‐mannoside (α‐MM), (Cat. No. M6882), Millipore Sigma, Burlington, MA, USA; Absolute ethanol (Cat. No. 64–17‐5), tryptic soy broth (Cat. No. 211825) and Todd Hewitt Broth (Cat. No. 249240), Becon Dickinson Laboratories, King of Prussia, PA, USA; Phosphate buffered saline (PBS) (Cat. No. BP2819), SNA‐I (Cat. No. NC1365364), phorbol 12‐myristate 13‐acetate (PMA) (Cat. No. 19‐144), Fisher Scientific, Pittsburgh, PA, USA; Prolong Gold antifade with DAPI (Cat. No. DU092202), Invitrogen, Carlsbad, CA, USA; Biotinylated *Galanthus nivalis agglu*tinin (biotin‐GNA) (Cat. No. B‐1245), FITC‐streptavidin (Cat. No. SA‐5001‐1), Vector Laboratories, Inc., Newark, CA, USA; Mycoplasma‐free immortalized human tracheal epithelial (HTE) cell line, which was established at the University of North Carolina at Chapel Hill, NC, USA [18], was provided by Dr. Reen Wu at the University of California at Davis, CA, USA [19]; Aldehyde dehydrogenase 2 activator 1 (Alda‐1) was provided by Dr. Maria Mochly‐Rosen at Stanford University at Stanford, CA, USA [20]. Ovine submaxillary mucin (OSM) was isolated as described [21].

### Live cell lectin stain of HTE cells

The immortalized HTE cells plated on the inserts of a 12‐well plate were treated with 50 mM EtOH for 40–48 h in a 37 °C 5% CO_2_ incubator, rinsed with PBS 3X, and then exposed to biotin‐GNA (20 μg·mL^−1^) in 1 mg·mL^−1^ BSA. After incubation at room temperature for 1 h, the cells were rinsed with PBS 3X and then exposed to FITC‐streptavidin (20 μg·mL^−1^) in 1 mg·mL^−1^ BSA. After 1 h, the cells were rinsed with PBS 3X followed by fixation in PBS buffered 5% formaldehyde for 10 min before treatment with antifade containing DAPI and examination under a confocal microscope. The fluorescence intensity at six random regions was measured by ImageJ and expressed as mean ± SEM fluorescence intensity/cell.

### Measurement of *Sp* and *Kp* adhesion to immortalized HTE cells

*Sp* was grown in Todd Hewitt Broth in a CO_2_ incubator (5% CO_2_) at 37 °C for 6 h while *Kp* was grown in tryptic soy broth in a shaking incubator (185 r.p.m.) at 37 °C for 18 h. Both bacteria were then pelleted by centrifugation (2000 ***g*** for 15 min at 4 °C), washed twice with phosphate‐buffered saline (PBS), and resuspended in PBS at a concentration of 1 × 10^9^ colony‐forming units (CFU)/mL for *Sp* and 5 × 10^9^ CFU·mL^−1^ for *Kp*.

HTE cells used for assay of *Sp* and *Kp* adhesion were cultured in Bronchial Life Medium containing supplements in 48‐well plates with or without alcohol treatment. Six wells per condition were used for each time point. The cultured cells were rinsed with PBS three times before exposure to 0.2 mL PBS containing 1 × 10^8^ CFU of *Sp* or 5 × 10^8^ CFU of *Kp* for 1 h. Then, the cells were rinsed with PBS three times to remove unbound bacteria. The number of bound *Sp* and *Kp* were then determined via standard serial dilution onto BD BBL™ Trypticase™ Soy Agar (TSA II™) with sheep blood or HiCrome *Klebsiella* Selective Agar respectively and then performing standard colony counts. To perform the assay of α‐MM inhibition of *Kp* adhesion to HTE cells, *Kp* were incubated with 0, 10, 30, or 50 mM α‐MM for 1 h before exposure to 50 mM alcohol‐treated cells. After 1‐h incubation, the CFU of bound *Kp* were analyzed as described above. To perform the assay of Alda‐1 inhibition of *Kp* adhesion to alcohol‐treated cells, HTE cells were cultured in 50 mM EtOH with and without 50 μM Alda‐1 for 44 h before exposure to *Kp* for 1 h. The CFU of bound Kp was analyzed as described above. The assays of 25 μg·mL^−1^ OSM and 50 μM Alda‐1 inhibition of *Sp* adhesion to 50 mM alcohol‐treated HTE cells were performed as described above.

### Statistics

Experimental results are expressed as mean +/− SEM. One‐way analysis of variance (ANOVA) models was used for statistical analysis. Pairwise group comparisons were made if the overall test was significant, adjusting for multiple comparisons with Tukey's methods. Data were considered significant if *P* ≤ 0.05.

## Results

### Exposure of HTE cells to EtOH induces high mannose N‐glycans terminated with α3mannose

**Fig. 1:**
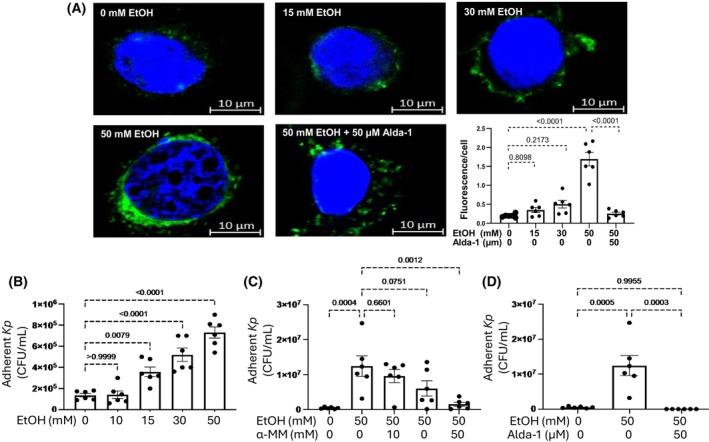
(A) Confocal immunofluorescence images of FITC‐GNA‐treated human tracheal epithelial (HTE) cells exposed to 15–50 mM EtOH and 50 mM EtOH + 50 μm Alda‐1. Adherence of *K. pneumoniae* to HTE cells that have been exposed to (B) 10–50 mM EtOH, (C) 50 mM EtOH ± 10–50 mm α‐methylmannoside (α‐MM), and (D) 50 mM EtOH ± 50 μM Alda‐1. Data are expressed as mean ± SEM (*n* = 6). ANOVA was used for statistical analysis.

Exposure of HTE cells to 15, 30, and 50 mM EtOH for 44 h induced high mannose N‐glycans terminated with α3mannose as detected with biotin‐GNA and FITC‐streptavidin (Fig. 1). Fifty mM EtOH induced a significant increase of high mannose N‐glycans terminated with α3mannose, which was inhibited by 50 μM Alda‐1(Fig. 1A).

### Exposure of HTE cells to EtOH enhances adhesion of Kp, which was inhibited by α‐methylmannoside or Alda‐1

Exposure of HTE cells to 15–50 mM EtOH significantly increased the adhesion of *Kp* (Fig. 1B). The increased adhesion of *Kp* to 50 mM EtOH‐treated HTE cells was inhibited by 30 or 50 mM α‐methylmannoside (Fig. 1C). The increased adhesion of *Kp* to 50 mM EtOH‐treated HTE cells was also prevented by blocking the formation of high mannose N‐glycans with 50 μM Alda‐1 (Fig. 1D).

### Exposure of HTE cells to EtOH enhances adhesion of Sp, which was inhibited by ovine submaxillary mucin (OSM) or Alda‐1

**Fig. 2:**
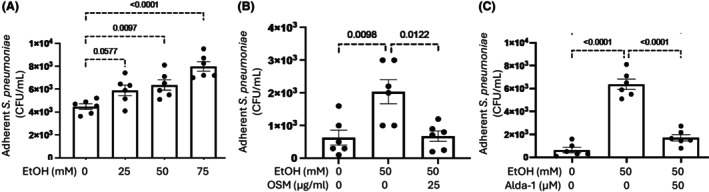
Adherence of *S. pneumoniae* to HTE cells that have been exposed to (A) 25–75 mM EtOH, (B) 50 mM EtOH ± 25 μg·mL^−1^ OSM, and (C) 50 mM EtOH ± 50 μM Alda‐1. Data are expressed as mean ± SEM (*n* = 6). ANOVA was used for statistical analysis.

Treatment of HTE cells with 25, 50, and 75 mM EtOH increased adhesion of *Sp* (Fig. 2A). The increased adhesion of *Sp* to 50 mM EtOH‐treated HTE cells was inhibited by treatment of *Sp* with sTn‐rich OSM (Fig. 2B) or by blocking the formation of α2‐6sialo mucin O‐glycans with 50 μM Alda‐1 (Fig. 2C).

## Discussion

Current study extends our recent discovery that alcohol acts via its metabolite acetaldehyde to induce α2‐6sialo mucin O‐glycans in human airway epithelial cells by inactivating giantin‐mediated Golgi targeting of glycosylation enzymes, which led to compromised immune function [16]. We have found that alcohol also induces high mannose N‐glycans, the same phenomenon reported in alcohol‐treated hepatocytes [15]. The α2‐6sialo mucin O‐glycans and high mannose N‐glycans induced by alcohol in HTE cells enhance adhesion of *Sp* and *Kp*, respectively. These alcohol effects can help explain the increased risk of bacterial pneumonia in heavy alcohol drinkers.

**Fig. 3:**
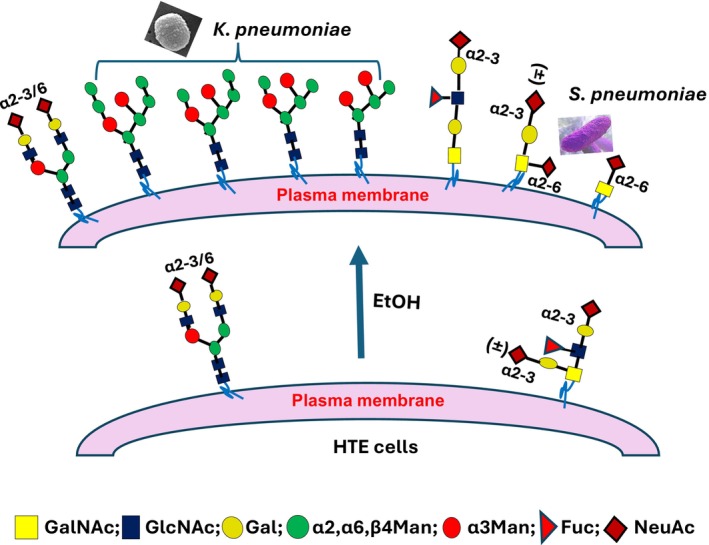
The mechanism of increased adhesion of pneumococcal bacteria in alcohol‐treated human tracheal epithelial cells. Alcohol treatment of human tracheal epithelial cells induces the formation of α2‐6sialo mucin O‐glycans and high mannose N‐glycans to promote adhesion of *S. pneumoniae* and *K. pneumoniae*, respectively.

Infections caused by many bacterial pathogens are initiated by adhesion to their glycan ligands present at the surface of the target cells [7]. In this study, we show that alcohol treatment of HTE cells enhances *Sp* and *Kp* adhesion by induction of α2‐6sialo mucin O‐glycans and high mannose N‐glycans terminated with α3mannoside, respectively. These interactions help retain these bacteria in the respiratory tract of heavy alcohol drinkers to promote pneumonia. Further, we have shown that α2‐6sialyl mucin O‐glycans kill U937 macrophages mediated by Siglec 7 [16]. Siglec 7 is a member of the inhibitory Siglec family present in many immune cells [10, 11, 22, 23]. Binding of Siglec 7 to its sialylated glycan ligands has been shown to suppress the cytotoxicity function of NK cells [22, 23, 24, 25, 26] and induces apoptosis of platelets [25]. By killing macrophages, the α2‐6sialyl mucin O‐glycans induced by alcohol help shield the bacteria in the respiratory tract from immune surveillance to help these bacteria survive and thrive. The effects of alcohol on the increase of pneumonia risk in people who are heavy alcohol drinkers are summarized in Fig. 3. Given that Siglec 7 is expressed in many immune cells [22, 23, 24, 25, 26] and Siglec 7‐expressing innate immune cells are highly functional immune cells [23], killing of Siglec 7‐expressing immune cells can have a significant negative impact on the host immune functions. For example, Siglec 7‐expressing natural killer cells are reduced in immune compromised patients with cancer [26], obesity [27], hepatitis B virus [28], hepatis C virus [29], and human immunodeficiency virus [30]. Understanding the extent of the effect of alcohol on overall cellular immunity is essential for developing therapy to treat alcohol toxicity. For example, treatment of airway epithelial cells exposed to alcohol with Alda‐1 can prevent formation of glycans, which compromise immunity and promote adhesion of pneumococcal bacteria. This compound may be a candidate as a potential therapeutic agent of pneumonia.

Heavy alcohol consumption increases the risk for acquiring pneumonia with symptoms manifested primarily in the alveoli [31]. The incidence of pneumonia in heavy alcohol drinkers is 39% higher than that in individuals without a history of heavy alcohol consumption [32]. The two mechanisms by which chronic heavy alcohol consumption increases pneumonia include reduced clearance of pathogens from the respiratory tract and impaired immune functions [31]. Altered glycans induced by alcohol play a key role in both mechanisms. As described in our recent publication [16], α2‐6sialo mucin O‐glycans induced by alcohol in human tracheobronchial epithelial cells kill macrophages via a siglec 7‐dependent mechanism, which supports reduced innate immunity in the alveoli of heavy alcohol drinkers who acquire pneumonia [4, 31]. The reduced clearance of pathogens from the respiratory tract can be attributed to suppression of cilia beating [31, 33] and diminished cough and gag reflex [31, 34]. Increased adhesion of *Kp* and *Sp* to cell surface high mannose N‐glycans and α2‐6sialo mucin O‐glycans induced by alcohol as described in our current study further impair patient's ability to remove these pathogens from the lungs. The pathogens that reach the alveoli are much harder to be cleared because they are furthest away from upper airways, which culminates in the development of more severe symptoms of pneumonia in this region of the respiratory tract. Further, for patients who suffer from persistent pneumonia, the phenomenon of intracellular pathogens needs to be considered. It is known that Group A *Streptococcus* can invade epithelial cells, endothelial cells, and macrophages and survive intracellularly [35]. Given that airway epithelial cells exposed to alcohol can produce α2‐6sialo mucin O‐glycans to protect these cells from being eliminated by immune cells, pathogens that enter these cells may survive intracellularly and prolong infections. This phenomenon and the effect of alcohol induction of altered glycans in epithelial cells and endothelial cells in the alveoli await examination.

Biofilm plays a key role in sustaining infection. Alcohol‐induced altered glycans can be present at the luminal surface of airway epithelial cells and the secreted glycoconjugates. The secreted glycoconjugates can help strengthen biofilm structure by connecting neighboring bacteria in the biofilm via the altered glycans. Further, these glycoconjugates can protect biofilm from immune surveillance by killing the immune cells that express siglec 7 via α2‐6sialo mucin O‐glycans. These possibilities await verification.

Alcohol toxicity is gender and body weight‐dependent [2, 3]. Current study shows that the adhesion of *Kp* to cultured human tracheal epithelial cells is significantly increased after exposure to 15 mM or 0.07% EtOH (*P* = 0.008). Also, 25 mM EtOH exposure increases adhesion of *Sp* (*P* = 0.058). Further, we recently showed that 30 mM ethanol‐treated human tracheal epithelial cells significantly increased the formation of α2‐6sialo mucin O‐glycans [16]. It is known that individuals with 0.08% or 17.4 mM blood alcohol concentration are considered impaired in driving and classified as driving under the influence (DUI) [36], which is a criminal offense. Table 1 summarizes the relationship between number of drinks consumed by 73 Kg males and females, which produce the blood alcohol concentrations [37, 38] equivalent to the alcohol concentrations, which enhance bacteria adhesion and killing of macrophages. It will take 2.5 drinks consumed by males and 2 drinks by females to produce 15 mM blood alcohol concentration. It will take 2.9 drinks by males and 2.3 drinks by females to produce 0.08% blood alcohol concentration. Therefore, the amounts of alcohol consumed by these males and females to promote infection of *Kp* are lower than those consumed by males and females to produce 17.4 mM DUI blood alcohol concentration. However, it will take > 4.3 drinks by males and > 3.4 drinks by females to increase adhesion of *Sp*. Since 30 mM alcohol induces α2‐6sialo mucin O‐glycans in human tracheal epithelial cells which can kill immune cells (16), males and females with 73 Kg body weight who consume 5 and 4 drinks, respectively may increase the probability of acquiring *Kp* and *Sp* infections by compromising immune functions and increasing adhesion of these two bacteria.

**Table 1:** Number of drinks consumed by 73 Kg males and females which produce BAC and their biological effects identified in this *in vitro* acute alcohol exposure study. Formula for calculation of the # of drinks which produce specific BAC [38, 39]: Number of drinks = BAC (%) × TBW/(100 × *F*_water_ × 14). Number of drinks, total amount of alcohol consumed divided by 14, which is the amount of ethanol per drink; BAC, blood alcohol concentrations, mg·100 mL^−1^ of blood; TBW, total body water, 41.9 L for 73 Kg males and 33.9 L for 73 Kg females; *F*_water_, water content of blood, 0.825% (w/v) in males and 0.838% (w/v) in females.

Number of drinks		BAC	
Males	Females	mM (g·100 mL^−1^)	Biological effects
2.5	2.0	15.0 (0.07%)	Significant increase in *Kp* adhesion to HTE cells
2.9	2.3	17.4 (0.08%)	DUI level
4.3	3.4	25.0 (0.11%)	Increased *Sp* adhesion to HTE cells
5.0	4.0	30.0 (0.14%)	Significant increase in the killing of macrophages

Alcohol induces not only high mannose N‐glycans and α2‐6sialyl mucin O‐glycans [15, 16] but also Tn, T, and α2‐3sialo mucin O‐glycans [16]. Some of these glycans can serve as the ligands of other pathogens, which can cause infections in the respiratory tract and gastrointestines as well as sepsis. For example, α2‐3 sialylated glycans can serve as a ligand for *Staphylococcus aureus* [39], *Haemophilus influenzae* [8], and *Mycobacterium tuberculosis* [8], and Tn as a ligand for *Clostridium difficile* [8]. Current findings can help guide future efforts to identify other disease‐causing bacteria, which benefit from the formation of these glycans induced by alcohol. This information can help stimulate efforts to develop strategies to prevent or alleviate alcohol toxicity.

## Conflict of interest

The authors declare no conflict of interest.

## Author contributions

PWC conceived the idea, coordinated the experimental design and execution of the confocal microscopy experiments, and wrote the manuscript. SD collected, analyzed, and graphed the data shown in Fig. 1A. DRS designed and supervised experiments related to bacterial binding and CFU analysis, and interpreted the results. He also performed data analysis and graphing, and edited the manuscript.

## Data Availability

The datasets underlying the article will be shared upon a reasonable request to the corresponding author.
